# Severity of SARS-CoV-2 infection in a hospital population: a clinical comparison across age groups

**DOI:** 10.1186/s13052-023-01485-w

**Published:** 2023-10-08

**Authors:** Chiara Rosazza, Laura Alagna, Alessandra Bandera, Arianna Biffi, Fabiana Ciciriello, Andrea Gramegna, Vincenzina Lucidi, Paola Giovanna Marchisio, Paola Medino, Antonio Muscatiello, Sara Uceda Renteria, Carla Colombo

**Affiliations:** 1 Paediatric Cystic Fibrosis Center, Fondazione IRCCS Ca’ Granda Ospedale Maggiore Policlinico, Milan, Italy; 2 Clinic of Infectious Diseases, Fondazione IRCCS Ca’ Granda Ospedale Maggiore Policlinico, Milan, Italy; 3 Department of Pathophysiology and Transplantation, University of Milan, Via Commenda 9, 20122 Milan, Italy; 4 Cystic Fibrosis Center, ‘Bambino Gesù’ Children’s Hospital, IRCCS, Rome, Italy; 5 Internal Medicine Department, Respiratory Unit and Regional Adult Cystic Fibrosis Center, Fondazione IRCCS Cà Granda Ospedale Maggiore Policlinico, Milan, Italy; 6 Pediatria Pneumoinfettivologia, Fondazione IRCCS Ca’ Granda Ospedale Maggiore Policlinico, Milan, Italy; 7 Virology Unit, Fondazione IRCCS Ca’ Granda Ospedale Maggiore Policlinico, Milan, Italy

**Keywords:** COVID-19, SARS-CoV-2 infection, Paediatric population, MIS-C, Outcome

## Abstract

**Background:**

Children tend to have milder forms of COVID-19 than adults, however post-acute complications have been observed also in the paediatric population. In this study, we compared COVID-19-related outcomes and long-term complications between paediatric and adult patients infected by SARS-CoV-2.

**Methods:**

The study is based on individuals enrolled from October 2020 to June 2021 in the DECO COVID-19 multicentre prospective study supported by the Italian Ministry of Health (COVID-2020–12371781). We included individuals with RT-PCR -confirmed SARS-CoV-2 infection, who were evaluated in the emergency department and/or admitted to COVID-dedicated wards. The severity of SARS-CoV-2 infection was compared across age groups (children/adolescents aged < 18 years, young/middle-aged adults aged 18–64 years and older individuals) through the relative risk (RR) of severe COVID-19. Severity was defined by: 1) hospitalization due to COVID-19 and/or 2) need or supplemental oxygen therapy. RR and corresponding 95% confidence intervals were estimated using log-binomial models.

**Results:**

The study included 154 individuals, 84 (54.5%) children/adolescents, 50 (32.5%) young/middle-aged adults and 20 (13%) older adults. Compared to young/middle-aged adults the risk of hospitalization was lower among paediatric patients (RR: 0.49, 95% CI: 0.32–0.75) and higher among older adults (RR: 1.52, 95% CI: 1.12–2.06). The RR of supplemental oxygen was 0.12 (95% CI: 0.05–0.30) among children/adolescents and 1.46 (95% CI: 0.97–2.19) among older adults. Three children developed multisystem inflammatory syndrome (MIS-C), none was admitted to intensive care unit or reported post-acute Covid-19 complications.

**Conclusions:**

Our study confirms that COVID-19 is less severe in children. MIS-C is a rare yet severe complication of SARS-CoV-2 infection in children and its risk factors are presently unknown.

## Background

COVID-19, the disease resulting from SARS-CoV-2 infection, has affected over 606 million people of whom approximately 6.5 million have died [1].

The spectrum of the disease in adults is well described, ranging from asymptomatic infection to respiratory failure and death. Disease severity increases with age, with consistently higher hospitalization and mortality rates in the older population [2–4]. Comorbidities also play an important role in determining the burden of the disease: the presence of underlying conditions, such as obesity, hypertension, malignancy and diabetes, is associated with higher disease severity, increased need of respiratory support and higher case-fatality rates [5, 6].

On the other hand, in the paediatric population, SARS-CoV-2 infection tends to be asymptomatic or mild, often limited to the upper respiratory tract, and persistent symptoms or long-term sequelae are rarely observed [7–9]. Information regarding long- COVID are particularly limited in paediatrics.

This study aims to compare COVID-19 outcomes, including possible long-term sequalae, across different age groups, in order to further characterize the burden of the disease in children.

## Materials and methods

The study is based on patients enrolled in the DECO COVID-19 project, a multicentre prospective study supported by the Italian Ministry of Health (COVID-2020-12371781), which involved 2 Italian tertiary care paediatric hospitals: 1) Fondazione IRCCS Ca’ Granda Ospedale Maggiore Policlinico in Milan (coordinating centre) and 2) Bambino Gesù Children's Hospital in Rome. The study was approved by the Ethics Committees of both hospitals and informed consent was obtained from the patient or parents/caregivers before inclusion in the study.

**Fig. 1:**
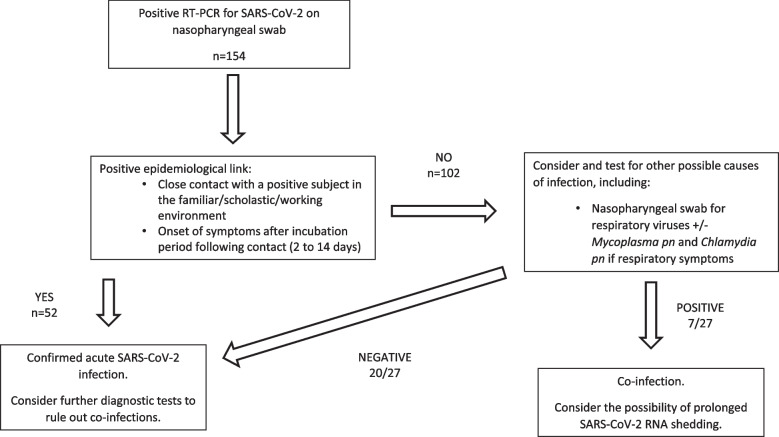
Diagnostic algorithm for symptomatic SARS-CoV-2 infection applied to the patients enrolled in the study

Between October 15, 2020 and June 30, 2021, all patients attending the emergency department and/or admitted to COVID-dedicated wards with a SARS-CoV-2 infection confirmed by real time polymerase chain reaction (RT-PCR) on a nasopharyngeal swab were enrolled. Demographic and clinical data were collected, including sex, age, body mass index (BMI), comorbidities, maintenance therapy, presence of acute symptoms and their duration, need of hospitalization, admission to intensive care unit (ICU), need of oxygen supplementation and non-invasive or invasive ventilation. In light of the fact that our study was based on PCR confirmed cases of SARS-CoV-2 infection, considering that viral shedding could continue long after the acute phase of the infection, particularly in the pre-vaccine era in which the study was set, we performed an accurate differential diagnosis in all positive patients presenting with acute symptoms suggestive of COVID-19. This included the assessment of an epidemiological link (close contact with subjects with proven active SARS-CoV-2 infection in the familiar/scholastic/working environment 2 to 14 days prior to symptom onset) and the exclusion of other possible causes of infection, by obtaining a nasopharyngeal swab for RT-PCR identification of the most common respiratory viruses, *Mycoplasma pneumoniae and Chlamydia pneumoniae* in patients with respiratory symptoms (Fig. 1).

We also gathered information on the complications related to SARS-CoV-2 infection, with a particular focus on multisystem inflammatory syndrome in children (MIS-C). Diagnosis of MIS-C was made according to the Royal College of Paediatrics and Child Health criteria [10]. Macrophage activation syndrome (MAS), a possible complication of MIS-C, was identified according to the diagnostic criteria formulated for MAS occurring in patients with systemic juvenile idiopathic arthritis [11].

Information on vital status at 6 months after the RT-PCR positive test was also registered.

Input of data was accomplished using the REDCap electronic data capture tool, hosted by Fondazione IRCCS Ca’ Granda Ospedale Maggiore Policlinico.

## Statistical analysis

We compared the severity of SARS-CoV-2 infection across the following three age groups: children/adolescents aged < 18 years, young/middle-aged adults aged 18–64 years and older individuals aged ≥ 65 years. Severity of COVID-19 was defined according to the following criteria: 1) need of hospitalization due to COVID-19 and/or 2) requirement or increased need of oxygen therapy.

Log-binomial models were used to estimate the relative risk (RR) of severe COVID-19 and corresponding 95% confidence intervals (CI) across age groups, using individuals aged ≥ 65 years as the reference category.

## Results

We enrolled 154 patients with RT-PCR-confirmed SARS-CoV-2 infection. Median age of the study population was 27 years (range: 1 month-89 years) and the study included 84 children/adolescents, 50 young/middle-aged adults and 20 older adults. The most frequently SARS-CoV-2 identified variants were lineage B.1.1.7 (42.4%) and B.1.177 (40.7%).

Table 1 shows the demographics and clinical characteristics of the study population, as well as the most frequently reported COVID-related symptoms. As expected, the presence of comorbidities increased with advancing age, with 9.5% of children/adolescents having at least one comorbidity compared with 44% among young/middle aged individuals and 90% of older individuals. In our series, the most frequent comorbidity in the paediatric age group was kidney disease, followed by chronic respiratory disease, whereas in the older age groups hypertension, cardiovascular disease and diabetes were the most frequently reported underlying diseases. Only one child (1.2%) presented with multiple comorbidities, as opposed to 16% of young adults and 60% of seniors. Paediatric patients with underlying diseases were almost invariably admitted to hospital wards (87.5% of cases), and 2/2 children suffering from a chronic respiratory condition required oxygen supplementation during the acute phase of COVID-19. None of the paediatric patients with comorbidities, however, required ICU admission or died.

**Table 1:** Characteristics of the study population by age group

	**Age group**		
	** < 18 years** **(*N* = 84)**	**18–64 years** **(*N* = 50)**	** ≥ 65 years** **(*N* = 20)**
Median age (years)	4 (range 0–17)	44 (range 19–64)	72 (range 65–89)
Male sex	47 (56.0)	24 (48.0)	10 (50.0)
No. of comorbidities^a^			
None	76 (90.5)	28 (56.0)	2 (10.0)
At least one	7 (8.3)	14 (28.0)	6 (30.0)
2 or more	1 (1.2)	8 (16.0)	12 (60.0)
Symptoms			
Asymptomatic	47 (56.0)	9 (18)	2 (10)
Fever	30 (35.7)	31 (62.0)	15 (75.0)
Fatigue	1 (1.2)	17 (34.0)	8 (40.0)
Dyspnea	2 (2.4)	17 (34.0)	13 (65.0)
Cough	11 (13.1)	24 (48.0)	8 (40.0)
Rhinitis	12 (14.3)	6 (12.0)	0
Anosmia/Dysgeusia	0	9 (18.0)	3 (15.0)
Diarrhea	5 (6.0)	5 (10.0)	2 (10.0)

Data are numbers (%)

^a^Including diabetes, hypertension, cardiovascular disease, kidney disease, liver disease, chronic obstructive pulmonary disease and cancer

The majority of children (56%) was asymptomatic, compared to 18% of young/middle-aged and 10% of seniors. The most frequent symptoms of SARS-CoV-2 infection were fever and cough in all age groups, while dyspnoea, anosmia and/or dysgeusia were almost specific of adult age.

Twenty-seven out of 154 patients, all belonging to the paediatric age group, were tested for possible coinfections, by performing RT-PCR viral, *Mycoplasma pneumoniae and Chlamydia pneumoniae* identification on a nasopharyngeal swab. At least a second viral agent in addition to SARS-CoV-2 was identified in 7 patients, of which 6 had Rhinovirus A/B/C and one had multiple viral coinfection (Rhinovirus and Adenovirus). No case of SARS-CoV-2 and *Mycoplasma pneumoniae* coinfection was identified in our series. Five of the 7 patients with documented viral coinfection required hospitalization and 4/7 needed oxygen therapy. All patients with multiple viral infections had a positive outcome, making a full recovery.

None of the paediatric patients had persistent symptomatic COVID-19 after 2 months from infection, while symptoms lasted longer than 2 months in 6 adults (3 in the 18–64 years age group and 3 aged ≥ 65 years).

Hospitalization was required in 27.4% of the children, 56% of young/middle-aged individuals and in 85% of older patients (Table 2).

**Table 2:** COVID-19 severity and outcome by age group

	**Age group**		
	** < 18 years** **(*N* = 84)**	**18–64** **(*N* = 50)**	** ≥ 65** **(*N* = 20)**
Hospitalization	23 (27.4)	28 (56.0)	17 (85.0)
ICU	0	1 (2.1)	3 (15.0)
Oxygen therapy	5 (6.0)	24 (48.0)	14 (70.0)
Mechanical ventilation	0	1 (2.0)	3 (15.0)
C-PAP	0	14 (28.0)	10 (50.0)
HFNC	3 (3.6)	14 (28.0)	9 (45.0)
ECMO	0	0	0
Death	0	1 (2.0)	2 (10.0)

Data are numbers (%)

*C-PAP* Continuous Positive Airway Pressure. *ECMO* Extra-Corporeal Membrane Oxygenation. *HFNC* High Flow Nasal Cannula. *ICU* Intensive Care Unit

**Fig. 2:**
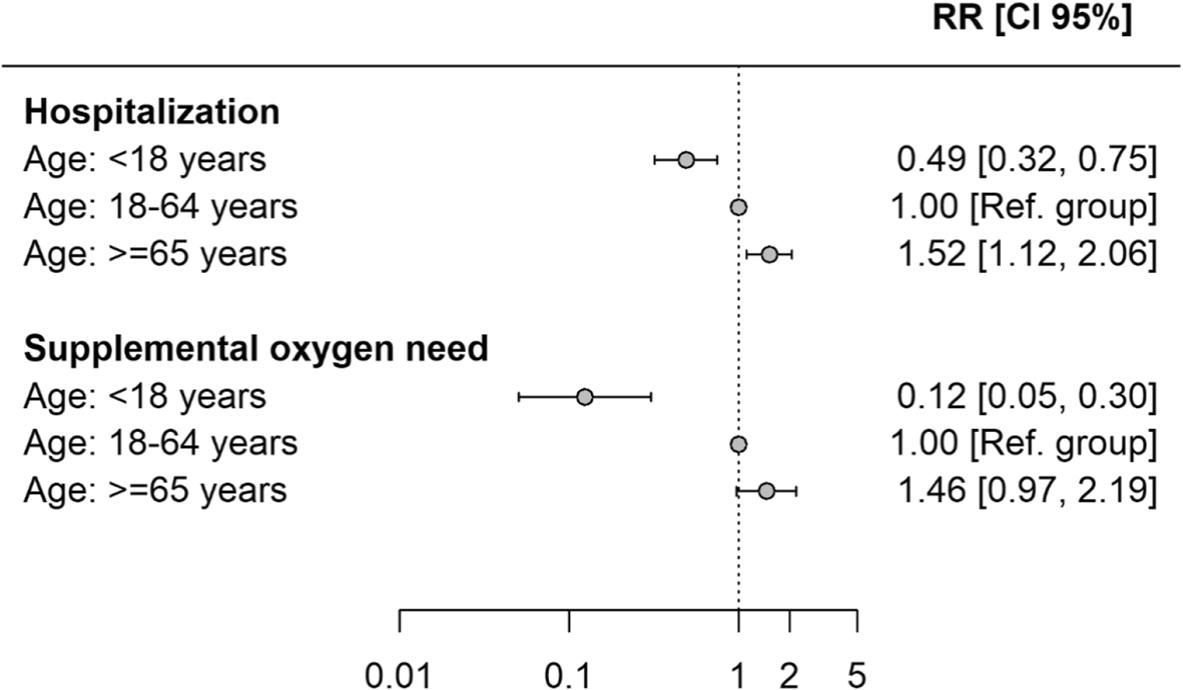
Relative risk (RR) and corresponding 95% confidence intervals (CI) of hospitalization and supplemental oxygen need according to age group (reference group: 18–64 years)

As compared to young/middle-aged, the risk of hospitalization was lower in the paediatric group (RR: 0.49) and higher among those aged ≥ 65 years (RR: 1.52) (Fig. 2). Only 6% of children needed oxygen therapy, whereas the percentage needing supplemental oxygen therapy was significantly higher in the young/middle-aged (48%) and older groups (70%). The RR of oxygen therapy was 0.12 among the paediatric population and 1.46 among individuals aged ≥ 65 years (Fig. 2). None of the paediatric patients required non-invasive respiratory support or mechanical ventilation, while 28% of the young/middle-aged and 50% of older adults needed non-invasive support and one patient aged 18–64 years and three patients older than 65 were intubated.

No intensive care unit admission or death due to COVID-19 was registered in the paediatric age group, while 4 adults (1 aged 58 years and 3 aged ≥ 65 years) required intensive care.

The length of stay was significantly shorter among paediatric patients (median, IQR: 8 days, 4–11), as compared to adults (15 days, 9–19.5 among young/middle aged adults and 14 days, 8–27 among older adults, *P* < 0.0003).

Three out of 84 paediatric patients were diagnosed with MIS-C. Clinical characteristics and therapeutic approach to these children are illustrated in Table 3. All patients were treated with intravenous immunoglobulin (IVIG), low-dose acetylsalicylic acid as a platelet aggregation inhibitor, proton-pump inhibitors (PPI) and antibiotic therapy. Two patients received adjunctive immunosuppressant treatment with intravenous high dose steroids. None of the patients had cardiac involvement in the context of MIS-C and no diagnosis of MAS was made in our case series. All children had recovered without sequelae at 6-month follow-up.

**Table 3:** Characteristics and outcome of the three children hospitalized for a diagnosis of MIS-C

Subject	Age (y)	Sex	Symptoms	Cardiac involvement (Yes/No)	sHLH/MAS	Blood exams	Therapy	Length of hospitalization (days)	Outcome
1	2	F	Fever, pharyngitis, anorexia, vomit, rash	No	No	CRP 10.4 mg/dL, ESR 73 mm/h, ferritin 207 ug/L, fibrinogen 609 mg/L, proBNP II 4032 ng/L, D-dimer 4549 ug/L, albumin 3.1 g/dL	Oral steroid, IVIg, ASA, PPI, cefotaxime	14	Recovery without sequelae
2	3	M	Fever, conjunctival hyperemia, anorexia	No	No	CRP 12.9 mg/dL, ferritin 536 ug/L, fibrinogen 333 mg/L, TnT 3 ng/L proBNP II 2998 ng/L, D-dimer 5326 ug/L	IVIg, ASA, PPI, ceftriaxone	11	Recovery without sequelae
3	8	F	Fever, diarrhea, vomit, pharyngitis, oral mucositis, conjunctival hyperemia, abdominal pain, headache	No	No	CRP 9.17 mg/dL, PCT 12.6 ug/L, ferritin 477 ug/L, fibrinogen 599 mg/L, TnT 3 ng/L proBNP II 118 ng/L, D-dimer 4911 ug/L, albumin 2.99 g/dL	IVIg, IV high-dose steroid, ASA, PPI, ceftriaxone, piperacillin/tazobactam	14	Recovery without sequelae

*CRP* C-reactive Protein, *PCT* Procalcitonin, *ESR* Erythrocyte sedimentation rate, *IL-6* Interleukin-6, *TnT* Troponin T, *proBNP II* Terminal pro-brain natriuretic peptide, *LDH* Lactate dehydrogenase, *IVIg* Intravenous immunoglobulin, *IV* Intravenous, *ASA* Acetylsalicylic acid, *PPI* Proton-pump inhibitors

At that time, none of the paediatric patients had died, as compared to 5 adults (2 in the 18–64 age group and 3 in the older group). All the deceased had 3 or more pre-existing diseases including diabetes, systemic hypertension, heart disease, liver disease, nephropathy and lung transplantation, which were often the main cause of death.

## Discussion

Our study confirms that COVID-19 is generally less severe in children, as indicated by lower rate of hospitalization and need of oxygen therapy. However, in a minority of children (~ 4% of our series) it may present with a severe phenotype, including MIS-C and MAS.

In our population, asymptomatic infections were more frequent in the paediatric age group than in adults: 56% of children, in fact, had no symptoms related to SARS-CoV-2 infection as compared to 18% in the young/middle-aged group and 10% in the older group. The rate of asymptomatic infection so far reported in children is quite variable, ranging from 15.5 to 65% [12–16]: this wide variability may be due to differences in the time of data collection, in geographical regions and in the different attitude towards screening diagnosis for contact tracing.

In agreement with previous reports, fever and respiratory symptoms were the most common clinical features both in the paediatric and adult population [17, 18], though symptoms in the paediatric age group were generally milder and limited to the upper airways. Anosmia/dysgeusia were reported almost exclusively in adult subjects [19–21].

The use of a diagnostic algorithm applied to the whole paediatric cohort enabled to assess the role of coinfections. Although documented only in a minority of patients exclusively belonging to the paediatric age, viral coinfections were associated with a greater burden of disease, including the need for hospitalization and oxygen supplementation. Though limited, data from our series suggest that coinfections may be associated with a more severe disease course also in children, and thus should be investigated in all patients with a diagnosis of SARS-CoV-2 infection.

Comorbidities, particularly diabetes, obesity and hypertension, are known risk factors for severe COVID-19 in adults [3, 4]. In our study population, comorbidities were, as expected, more frequent in the young/middle-aged and in the older age groups and this may likely explain the greater COVID-19 severity among adults. The eight children with comorbidities identified in our series, however, had a more severe disease course, as opposed to paediatric patients with no underlying disease. Recent meta-analyses have highlighted that the presence of comorbidities increases the risk of severe COVID-19 and associated mortality also in children, and further prospective studies are needed to better characterize the impact of underlying conditions on the clinical course of paediatric COVID-19 [22–24].

The length of hospital stay was significantly lower in the paediatric group, as compared to the young/middle aged and older groups; this finding is consistent with a retrospective analysis of subjects from the early phase of the pandemic conducted in China, which documented significantly shorter hospital stay for COVID-19 in children than in adults [25]. None of the children included in our study, required intensive care or died because of SARS-CoV2 infection, confirming previous reports of low requirement for intensive treatments and low mortality rate in the paediatric population [26, 27]. A meta-analysis of paediatric COVID-19 cases (0 – 17 years), occurring over the period from August 2020 to August 2021 in the United States, identified a low percentage of patients requiring invasive mechanical ventilation (0 to 3%) and a death rate of 0.4% among hospitalized children [28].

None of our paediatric patients suffered from long-COVID, according to the National Institute for Healthcare Excellence definition, which includes both ongoing (4 to 12 weeks) and post-COVID-19 (≥ 12 weeks) symptoms [29]. However, the actual risk of persistent symptoms following acute COVID-19 in children is uncertain, in addition features of long-COVID are poorly characterized in this age group [30]. Long-term sequelae related to COVID-19 were described more rarely in children than in adults, although recent systematic reviews have reported a consistent number of paediatric long-COVID cases (with a highly variable prevalence depending on the considered case series, ranging from 4 up to 66%), highlighting how the presence of long-lasting COVID-19 manifestations should be investigated also in the paediatric population [29–32]. Case series reported by Brackel emphasize the nonspecific and broad clinical manifestations seen in post‐COVID complaints [30]. Notably, neuropsychiatric symptoms are the most frequent long-term complications in paediatric patients with a history of SARS-CoV-2 infection, particularly among pre-adolescents and adolescents, with mood and sleep disorders and fatigue being the most frequent complaints [32]. However, it is often difficult to assess whether these symptoms are a direct consequence of COVID-19 or should be more appropriately attributed to pandemic-induced stress and its related restrictions [33, 34].

MIS-C is a multisystem inflammatory syndrome affecting patients aged 0 to 19 years and related to SARS -CoV-2 infection either at the time of diagnosis or in the preceding 2 to 6 weeks. The most frequent clinical features of MIS-C include fever, mucocutaneous findings, cardiac involvement (myocardial dysfunction, cardiac conduction abnormalities and shock), gastrointestinal symptoms, lymphadenopathy, and neurological manifestations. The exact incidence of this condition is unknown, and at present it is considered a rare complication of SARS-CoV-2 infection [35]. Due to its possible cardiac involvement, MIS-C is a potentially life-threatening condition in the absence of a timely treatment with immunosuppressant agents (IVIG and high dose IV steroid pulses represent the first line therapeutic approach) [36–38]. Our study identified a total of 3 cases of MIS-C (all younger than 10 years) out of 84 paediatric patients with proven SARS-CoV-2 infection. All patients were successfully treated with IVIG and high dose IV steroids and none required intensive care, probably as a result of a timely diagnosis and early treatment initiation in tertiary care paediatric centers.

The main limitation of our study is the relatively small size of the enrolled population. This is mainly due to the fact that the present analysis was conducted in the context of a wider project, sponsored by the Italian Ministry of Health. Patients with cystic fibrosis, bronchiectasis and lung transplantation with acute SARS-CoV-2 infection were enrolled over a short period of time and their severity of disease and outcome was compared with the general population. In addition, since the study population was enrolled before the onset or at the earliest stage of the vaccination campaign in our country, we could not evaluate the impact of SARS-CoV-2 vaccination on disease severity and outcome. Finally, data were collected in the pre-Omicron phase of the pandemic, when circulating variants caused a more severe disease than the one secondary to Omicron.

However, the fact that we considered only RT-PCR confirmed cases, the search for coinfection and the evaluation of their impact on the severity of disease, the use of objective parameters to assess disease severity and the availability of a 6-month follow up for all the included patients represent significant strengths of our study.

## Conclusions

This study confirms that COVID-19 is generally less severe in children than in adults. While fever and respiratory symptoms are the most common clinical manifestations in all age groups, symptoms in the paediatric population tend to be milder and often related to inflammation limited to the upper airways, without long-term sequelae. MIS-C is a rare yet severe complication of SARS-CoV-2 infection in children, whose risk factors need to be further defined.

Although the SARS-CoV2 pandemic is currently in a resolving phase, the expertise acquired in the assessment of patients in a pandemic setting and the diagnostic algorithm we have used for the present study could represent a useful tool in the management of possible future infectious outbreaks.

## Data Availability

All data generated or analysed during this study are included in this published article.

## Abbreviations

RT-PCR: Real time polymerase chain reaction
BMI: Body mass index
ICU: Intensive care unit
MIS-C: Multisystem inflammatory syndrome in children
MAS: Macrophage activation syndrome
RR: Relative risk
CI: Confidence intervals
IVIG: Intravenous immunoglobulin
PPI: Proton-pump inhibitors
